# Histamine plasma levels from dietary histidine/histamine intake correlate with CGRP in trigeminal tissues

**DOI:** 10.1186/s10194-025-02178-x

**Published:** 2025-11-13

**Authors:** Fernando de Mora, Mária Dux, Birgit Vogler, Annette Kuhn, Jana Schramm, Karl Messlinger

**Affiliations:** 1 Department of Pharmacology, Therapeutics and Toxicology, Universidad Autónoma de Barcelona, Barcelona, Spain; 2 Department of Physiology, University of Szeged, Szeged, Hungary; 3 Institute of Physiology and Pathophysiology, Friedrich-Alexander-University Erlangen-Nürnberg, Erlangen-Nürnberg, Germany

**Keywords:** Calcitonin gene-related peptide, CGRP, Headache, Histamine intolerance, Migraine, Trigeminal sensitization

## Abstract

**Background:**

Trigeminal afferents innervating the meninges are likely involved in the generation of headaches and migraine. A major proportion of these afferents can release calcitonin gene-related peptide (CGRP) upon stimulation. Several substances, among them histamine, are known to induce headaches and trigger migraine. In addition to endogenous histamine, high dietary intake of histidine or histamine, or impaired histamine degradation in the gut, can lead to symptoms of histamine intolerance such as headache. However, it remains unclear whether and how dietary histamine impacts the trigeminal system, particularly trigeminal afferents releasing CGRP.

**Methods:**

In mice supplied with high dietary histidine/histamine, CGRP content in different tissues and CGRP release from the dura mater was utilized as a measure of potential histamine-induced sensitization of trigeminal afferents. After 19–32 days of feeding mice with a diet containing high histidine and histamine levels, CGRP concentrations were measured in plasma as well as in homogenized samples of ileum, trigeminal ganglia, spinal medulla and cerebellum, and compared with those from a control group fed a standard chow. CGRP content and release data were correlated with previously analysed histamine content and release data. Likewise, CGRP release from the cranial dura mater stimulated with the TRPV1 receptor agonist capsaicin was determined using our validated hemisected cranial preparation.

**Results:**

Mice fed high histamine-histidine diets exhibited an increased trigeminal ganglion CGRP concentration that correlated with diet-derived plasma histamine levels. The capsaicin stimulated CGRP release from the dura mater was higher in animals supplied with high histidine/histamine diet compared to control animals. An exponentially higher amount of dietary histidine likely converted into histamine in the gut appeared to be the main contributor to modulating CGRP levels. Separating for sexes and normalizing to the body weight of the animals, this difference was attributable to male mice.

**Conclusions:**

High dietary histidine or histamine, by elevating plasma histamine levels, cause increased CGRP concentrations and ongoing CGRP release from peptidergic afferents in trigeminal tissues. Exogenous histamine-induced sensitization of trigeminal afferents may facilitate headache generation and contribute to trigger migraine attacks.

**Supplementary Information:**

The online version contains supplementary material available at 10.1186/s10194-025-02178-x.

## Introduction

Histamine is a biogenic amine synthesized from histidine in the epidermis and nervous system, where it functions as both a tissue hormone and neurotransmitter [1, 2]. It is stored in high concentrations in mast cells, basophils, and chromaffin-like cells and can be released via IgE-, complement-, or compound 48/80-induced degranulation [3, 4]. In addition, histidine is converted into histamine by bacteria within the gastrointestinal system [1].

Histamine has been implicated in migraine pathophysiology, although evidence is primarily preclinical and clinically inconclusive [5, 6]. Mast cell degranulation contributes to neurogenic inflammation, experimentally demonstrated in the rodent dura mater, and has been linked to migraine mechanisms [7–9]. Histamine released from dural mast cells may activate meningeal afferents [10] and the trigeminal nociceptive system [11], leading to CGRP-mediated vasodilation and increased meningeal blood flow [12, 13]. High CGRP levels can also induce mast cell degranulation [7, 14], and the resulting histamine may further enhance vasodilatation [15].

During migraine attacks, CGRP released from meningeal tissues is a key driver of increased blood flow [16, 17]. Elevated CGRP levels have been detected in venous outflow [18], saliva [19], and tear fluid [20], and they normalize with effective treatment or after attack resolution [21, 22]. CGRP is now recognized as a causative agent in migraine pain [23], though its nociceptive effects likely require prior afferent sensitization [24–26]. Therapeutics targeting CGRP pathways, including gepants [30] and monoclonal antibodies against CGRP (e.g., fremanezumab) or its receptor (e.g., erenumab), have proven effectivity in migraine therapy and prevention [27–31]. Histamine also appears to contribute independently to migraine. Infusion of histamine can provoke migraine-like attacks, which are blocked by H1 antagonists [32]. Patients with both migraine and allergies show elevated plasma histamine levels during attacks [33], and inhibiting mast cell activity reduces CGRP-induced firing of meningeal sensory neurons in rats [34].

Beyond a role of endogenous histamine produced systemically or neurologically, we hypothesized that high histamine concentration in the gut may not only influence local nerve fibres, but may also diffuse into the systemic circulation to contribute to both CGRP release and migraine development [2]. Subsequent analysis revealed that dietary histamine and/or histidine modulated plasma histamine levels [3]. Notwithstanding the mechanism, dietary histamine has been described to cause migraine in patients exposed to food containing high histamine levels [4–6]. A few studies claim that migraine symptoms may be aggravated in individuals with histamine intolerance (HIT) associated to impaired exogenous histamine degradation, possibly due to a deficiency of diaminooxidase (DAO), the main enzyme involved in the metabolism of histamine [7, 8]. Consequently, a role for the histamine-degrading enzyme DAO in migraine prevention has been advocated [7, 9]. Therefore, further to well-described downstream CGRP-to-histamine events [10, 11], there may be an upstream histamine-to-CGRP connection in migraine that would support the theory of an exogenous histamine-induced sensitizing effect of trigeminal afferents in the dura mater finally promoting an increase in CGRP release.

This study forms the core of a broader project investigating the effects of high dietary histidine/histamine in mice on the histamine and CGRP content in different non-neural and neural tissues as well as histamine and CGRP release from the cranial dura mater. In a recent paper we have published the dietary conditions, food consumption, body weight and histamine content and release by comparing cohorts of mice supplied with zero and high concentrations of histamine in the diet [3]. Here we present our findings on tissue CGRP concentration and release, and correlate them with the previously published results on histamine concentration and release. In particular, given the central role of the trigeminal ganglion as a source of CGRP in migraine, we hypothesized that diet-derived plasma histamine may reach the trigeminal ganglion and other neural structures to promote CGRP production. To test this, we conducted a correlation analysis between plasma histamine levels and tissue CGRP content. Finally, we discuss possible mechanisms involved in a functional association between exogenous histamine from the diet and CGRP concentrations in extraneural and neural tissues as well as CGRP release from the dura mater.

## Methods

### Animals

 Young adult C57BL/6 mice (males 25–27 g, females 20–22 g body weight) were purchased from Charles River (Sulzfeld, Germany) were individually ear-marked, held in groups of 3 male or female animals and fed with standard raising food pellets (Ssniff GmbH, Soest, Germany) and water ad libitum were used for the experiments. Animal housing and all experiments were carried out according to the German guidelines and regulations of the care and treatment of laboratory animals and the European Communities Council Directive of 24 November 1986 (86/609/EEC), amended 22 September 2010 (2010/63/EU).

### Low and high histidine/histamine diet feeding 

After acclimatization for about one week, the animals were weighed and randomly allocated to control and test groups. One control group was continuously supplied with standard Altromin C1072 with no added histamine and low amounts of histidine (4.675 mg/kg) (Altromin low-histamine control group), the test group was supplied with Altromin C1072 containing additional 3 g/kg histamine (Altromin high-histamine test group) for 22–32 days (Table 1). The diet from Altromin GmbH (Lage, Germany) was chosen based on previous literature data [12]. Another control group of animals was continuously supplied with standard raising food from Ssniff containing 5.8 g/kg histidine (Ssniff low-histamine control group), and the corresponding test group was supplied with Ssniff standard food plus 9 g/kg histamine (Ssniff high histamine test group) for 19–22 days (Table 1). During the feeding periods all animals were inspected and weighed every day. For technical reasons, the final experiments had to be spread over several days, so that the feeding time was not exactly the same in all animals.

**Table 1:** Histamine and histidine content in control and high-histamine diets administered to mice

	Control group (standard diet)	Test group (high histamine diet)
Altromin^®^	*Histamine*: zero *Histidine*: 4.675 mg/kg	*High Histamine*: + 3 g/kg *Histidine*: 4.675 mg/kg
Ssniff^®^	*Histamine*: zero *Histidine*: 5.8 g/kg	*High Histamine*: + 9 g/kg *Histidine*: 5.8 g/kg

### Organ/tissue preparations

 Animals were deeply anaesthetized with inhalation of isoflurane followed by i.p. injection of thiopental until spontaneous ventilation and heart beat stopped, then quickly thoracotomized and exsanguinated by aspiration of blood from the left ventricle using an EDTA-coated syringe with a 22G needle. The blood was centrifuged and the supernatant plasma was taken off and deep-frozen. The head including the cervical segments was separated from the body and skinned. The occipital bone was opened and the medulla together with the cerebellum was separated from the cerebral cortex. The medulla oblongata at a length of 2 mm and the cerebellum were separated and quickly frozen. Then the skull was divided in the sagittal plain using a sharp blade and the cortical halves were removed and immersed in SIF (see below). Care was taken not to touch the dura mater lining the skull during the preparation. The trigeminal ganglia of both of the hemi-skulls were dissected immediately after the release experiments (see below) and frozen. Some animals were laparatomized and about 1 cm of the ileum was dissected and frozen. After defrosting, the tissue samples were dipped on a filter paper to remove adhering fluid, weighed and heated with 0.o1% perchloric acid at 95 °C. Then the samples were homogenized using a custom-made vortex, again heated in 0.o1% perchloric acid at 95 °C, centrifuged and neutralized with NaOH. 100 µL of the supernatant were taken off for determining the CGRP content (see below).

### CGRP release experiments 

The hemi-skulls with the lining dura mater were washed in neutral buffer synthetic interstitial fluid (SIF). SIF is composed of (in mM): 108 NaCl, 3.48 KCl, 3.5 MgSO4, 26 NaHCO3, 11.7 NaH2PO4, 1.5 CaCl2, 9.6 Na-gluconate, 5.55 glucose and 7.6 sucrose, adjusted to pH 7.4 with carbogen gas. The hemi-skulls were mounted on a water bath at 37 °C and filled with 200 µL SIF two times for 5 min, then with a solution of the transient receptor potential vanilloid 1 (TRPV1) receptor agonist capsaicin (500 nM) in SIF for 5 min to stimulate CGRP release from meningeal afferents. From each solution, samples of 100 µL were taken off and supplemented with 25 µl enzyme-linked immunoassay (EIA) buffer (Bertin Pharma/SPIbio, Montigny le Bretonneux, France) containing peptidase inhibitors. All fluid samples were frozen until CGRP measurements by ELISA, which was usually performed within one week. Following the CGRP release procedure, every hemi-skull was again washed with SIF and subjected to the histamine release procedure induced by compound 48/80, as described in our previous publication [3].

### CGRP ELISA

 After defrosting, the CGRP concentration in each sample was measured by an ELISA method (CGRP ELISA A05481, Bertin Pharma/SPIbio, Montigny le Bretonneux, France), which is based on a double-antibody sandwich technique with monoclonal capture antibodies fixed to plastic wells and soluble tracer antibodies specifically binding the CGRP molecule at different sites. The tracer antibodies are conjugated with acetylcholine esterase (AchE) that converts Ellman’s reagent to a yellow product, the absorbance of which is measured at a wavelength of 405 nm using a spectrophotometer (Opsys MR, Dynex Technologies, Denkendorf, Germany). The intensity of this colour, which is proportional to the amount of anti-CGRP tracer bound to the CGRP captured in the well and hence proportional to the amount of free CGRP in the samples, is compared with that of graded CGRP standard solutions. The ELISA detects both α- and β-CGRP with a lower limit of 2 pg/mL, and has no cross-reactivity with histamine.

### Calculation and statistics 

The biometry was based on data of our previous measurements of histamine release from the dura mater and histamine concentrations in different tissues comparing control and high-histamine diets as used in the present study [3]. CGRP concentrations in the blood plasma were calculated as pg/mL and in the homogenized samples as pg/mg tissue, considering the added volume of EIA buffer. Statistical analysis was performed using Statistica 13 software (StatSoft, USA). Data were compared with factorial ANOVA extended by Tukey’s HSD post-hoc test using the factors food (Altromin vs. Ssniff), histamine diet (high histamine vs. low histamine control diet) and sex (male vs. female). CGRP release data were expressed as pg/mL superfusion fluid and, in addition, re-calculated normalized to the body weight of each animal and expressed as pg/g body weight to compensate for the different size of the dura mater. Data were compared with repeated measures ANOVA extended by Tukey’s HSD test using the factors repetition (SIF1, SIF2, Capsaicin), food, histamine diet and sex. In an extended analysis, using Pearson’s product moment correlation (Statistica 13 software), the CGRP concentration and the CGRP release data were compared with the respective histamine data from same experiments, which have previously been calculated and recently published [3]. Data are shown as means ± standard deviation (SD) or standard error of means (SEM). A probability level of *p* < 0.05 was regarded as statistically significant.

## Results

### General condition of mice

For this study, 60 mice of both sexes at equal numbers were used. Details concerning the condition of animals such as changes in body weight have been reported in our recently published paper [3]. Experimental groups of mice were fed diets bearing from no histamine to high histamine contents. One cohort of 36 animals was supplied either with the Altromin control or with the high-histamine diet, another cohort of 24 animals with the Ssniff control or the high-histamine diet. After switching from standard control food to diets with high histamine, animals consumed less food for 1–3 days, but thereafter all animals gained weight at a similar rate, males more than females [3]. No abnormality in appearance, physical health or behavior was detected during the feeding periods in any of the animals of the low and high histamine diet groups.

### CGRP levels in low and high histidine/histamine diet-fed mice

**Fig. 1:**
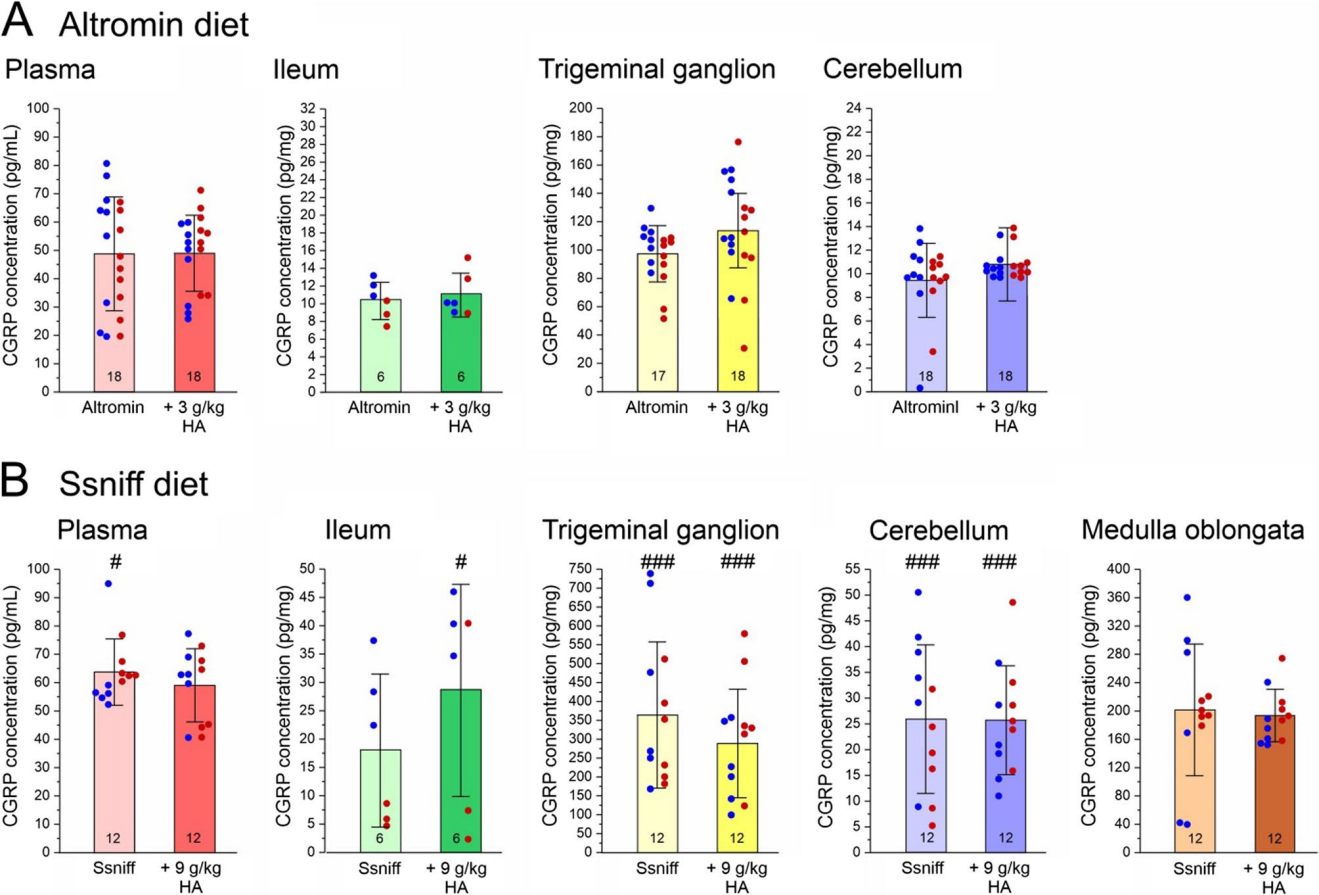
CGRP concentration in mouse plasma (pg/mL), homogenized ileum, trigeminal ganglion, cerebellum and medulla oblongata (pg/mg) in animals fed low or high histamine (HA) diets, i.e. Altromin with 3 g/kg histamine for 22–32 days **A** or Ssniff with 9 g/kg histamine for 19–22 days **B**. Since there was no significant sex difference, data of both sexes are pooled, but single values are shown as dots, blue for male and red for female animals. The CGRP concentration was significantly higher in tissues of animals fed Ssniff compared to Altromin (#, *p* < 0.05; ###, *p* < 0.0005), but there was no difference between corresponding control diets and high-histamine diets. Sample numbers are shown in the bars. All data are means ± SD

The CGRP concentration was determined in blood plasma, ileum and neural tissues (trigeminal ganglion, cerebellum and medulla oblongata) in mice of the control and the test groups. Generally, CGRP concentrations seemed to be higher in samples of Ssniff diet compared to Altromin diet fed mice. Factorial ANOVA with the factors “diet” (Altromin vs. Ssniff), “histamine” (control vs. test groups) and “sex” (males vs. females) indicated significant differences in the CGRP concentration between animals fed Altromin and animals fed Ssniff diet in plasma (*F*_1,52_ = 9.67, *p* < 0.005), trigeminal ganglion (*F*_1,51_ = 62.29, *p* < 0.0005), cerebellum (*F*_1,52_ = 65.65, *p* < 0.0005) and ileum (*F*_1,16_ = 14.45, *p* < 0.005), but there was no sex difference, apart from the ileum (*F*_1,16_ = 12.51, *p* < 0.005). In Fig. 1, data from animals supplied with Altromin and Ssniff are separately displayed. ANOVA extended by the Tukey HSD test showed no difference in the CGRP concentrations between corresponding control and high-histamine diets in any of the tissues. However, there are significant differences (*p* < 0.05) in the CGRP plasma levels between the control groups, CGRP concentrations in ileum between the high-histamine groups and highly significant differences (*p* < 0.0005) in neural tissues between Altromin and Ssniff fed mice. All significance values between specific groups are shown in Supplementary Table 1.

### Correlation of CGRP and Histamine concentration in different tissues

Data from same experiments, in which CGRP content and histamine content was assessed, were subjected to correlation studies. There was a tendency towards a positive correlation between CGRP and histamine in plasma as well as in ileum and a tendency of a negative correlation in the trigeminal ganglia and the cerebellum, but not in the medulla (Table 2). Separating the data for sexes, values were similar except for the female medulla, in which a significant negative correlation was calculated (*, see Table 2). When separating the data for high histamine diet and control diet, the above tendencies were largely conserved (Table 2). Likewise, there was no clear difference when data were separated according to Altromin and Ssniff diet (not shown).

**Table 2:** Comparison of CGRP and Histamine concentrations in different tissues calculated by product moment correlation (*n*, number of samples; *r*, pearson’s product moment correlation coefficient; *, *p* < 0.05)

	All		Males		Females		High HA diet		Control diet	
	*n*	*r*	*n*	*r*	*n*	*r*	*n*	*r*	*n*	*r*
Plasma	72	0.22	36	0.25	36	0.19	36	−0.12	66	0.23
Ileum	23	0.15	11	0.23	12	−0.16	11	−0.31	18	0.44
TGs	71	−0.2	35	−0.33	36	−0.13	36	−0.14	65	−0.20
Medulla	36	−0.05	18	−0.03	18	−0.80*	18	0.11	30	−0.17
Cerebellum	71	−0.23	36	−0.25	35	−0.29	36	−0.15	65	−0.25

### Correlation of CGRP concentration in different tissues with plasma histamine levels

Assuming that plasma histamine levels may influence the CGRP concentration in neural and extraneural tissues, we compared the corresponding data from our previous histamine measurements with the current CGRP measurements [2]. There was a positive correlation between plasma histamine levels and CGRP concentration in the trigeminal ganglion, establishing a direct link between circulating histamine and CGRP production by trigeminal ganglion afferent neurons, as shown in Fig. 3. Separating the data for sexes, values were similar and significant either in males or females. A positive correlation existed also between plasma histamine level and CGRP concentration in the medulla oblongata but not in the ileum (Table 3). When separating the data for high-histamine diet and control diet (irrespective of Altromin or Ssniff diets), these results were conserved only in the samples of animals fed with the control diets (Table 3).

**Table 3:** Comparison of plasma Histamine levels and CGRP concentrations in different tissues calculated by product moment correlation (*n*, number of samples; *r*, pearson’s product moment correlation coefficient; *, *p* < 0.05)

	All		Males		Females		High HA diet		Control diet	
	*n*	*r*	*n*	*r*	*n*	*r*	*n*	*r*	*n*	*r*
TGs	71	0.32*	34	0.30	36	0.35*	36	−0.06	35	0.48*
Medulla	36	0.45*	18	0.59*	18	0.25	18	−0.20	18	0.64*
Cerebellum	72	0.10	35	0.30	36	−0.09	36	−0.25	36	0.27
Ileum	24	0.09	12	0.51	12	−0.31	12	−0.07	12	0.46

### CGRP release from the dura mater

**Fig. 2:**
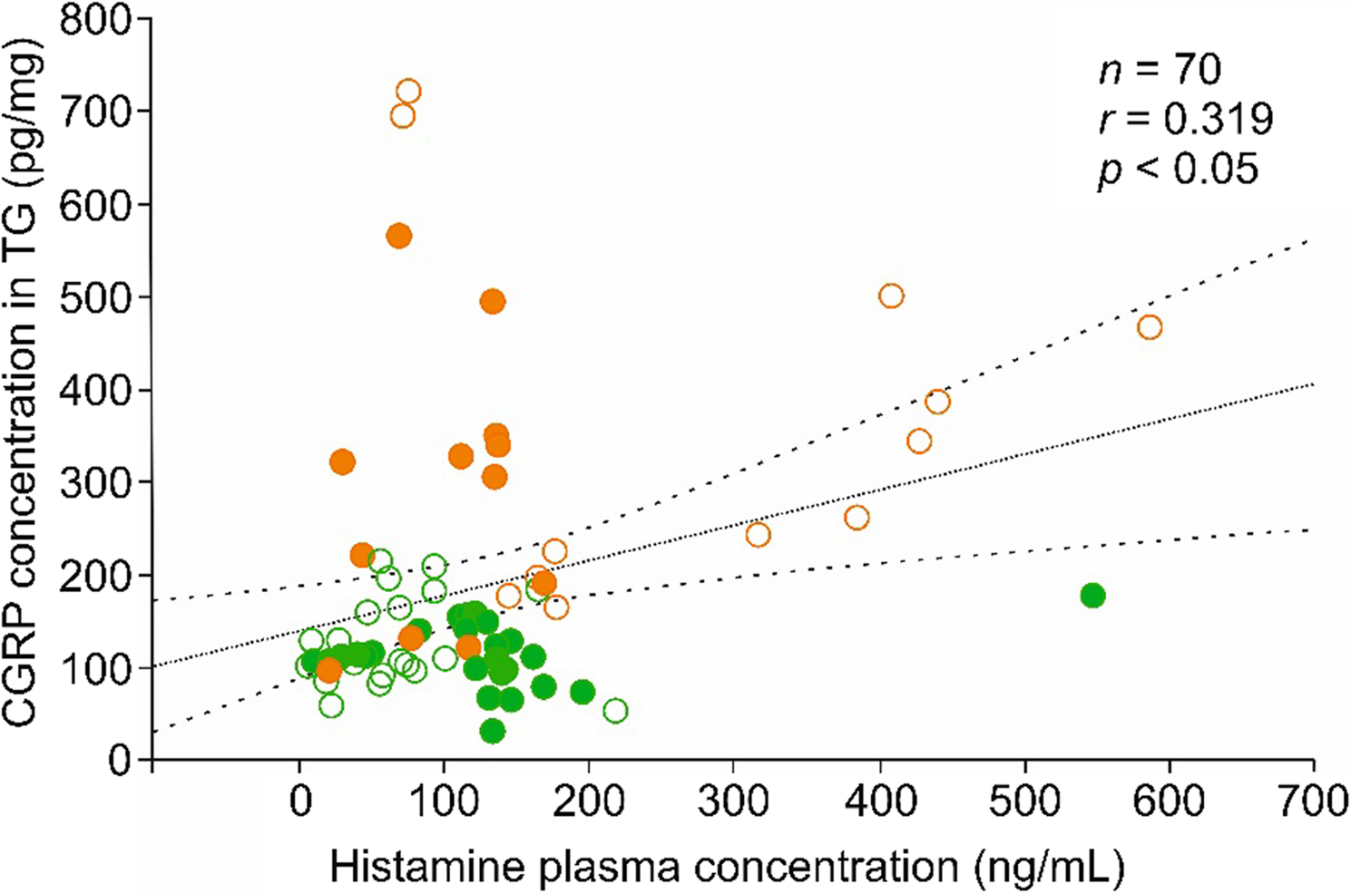
Linear correlation of the CGRP concentration in trigeminal ganglia (TG) with the histamine plasma concentration. Green symbols denote preparations from animals fed Altromin, orange symbols from animals fed Ssniff; open circles show preparations from control animals, filled circles from animals with high-histamine (3 g/kg or 9 g/kg) diets. Broken lines confine the 95% confidence interval

**Fig. 3:**
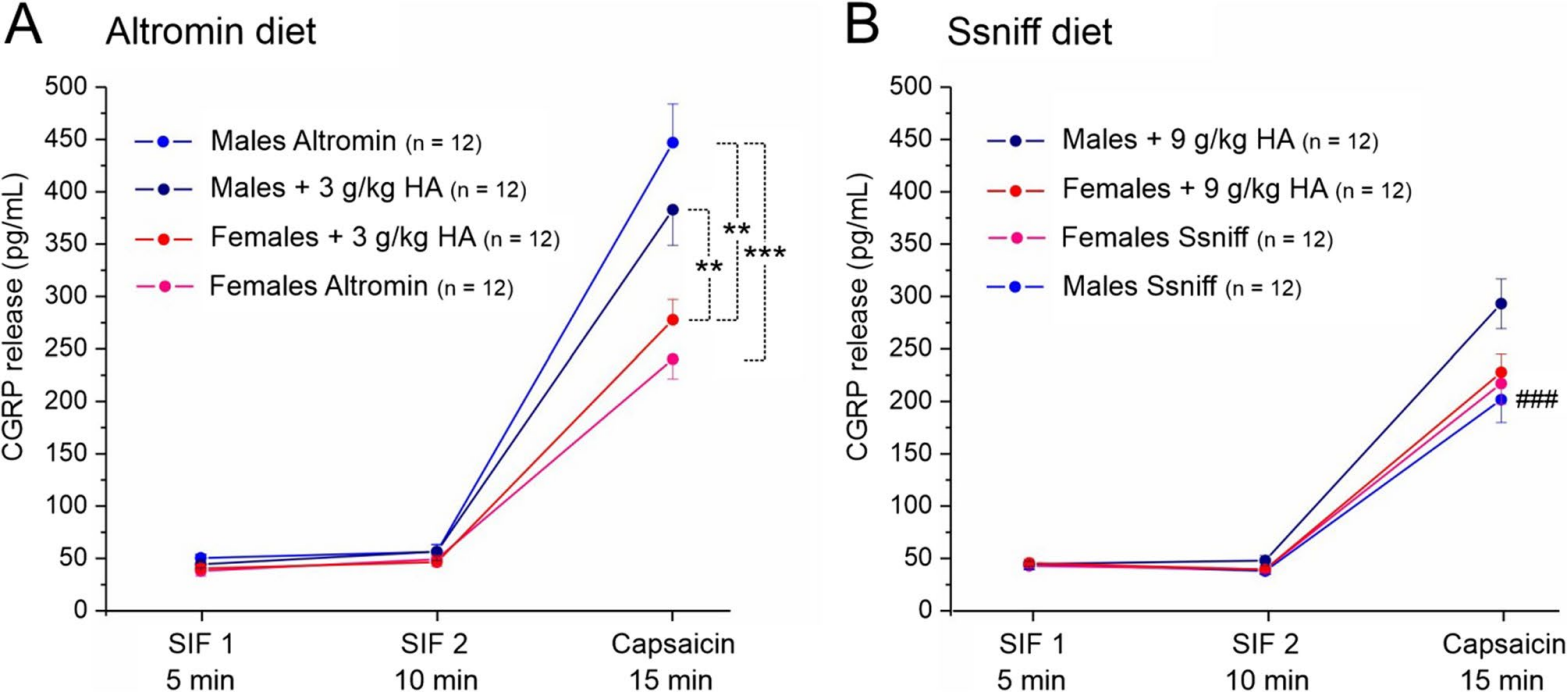
CGRP concentration in the superfusion solution of the hemisected skull preparations from animals fed Altromin control diet or Altromin plus 3 g/kg histamine **A** and Ssniff control diet or Ssniff plus 9 g/kg histamine **B**. Values are compared with repeated measures ANOVA followed by the Tukey post-hoc test and shown as means ± SEM; **, *p* < 0.005; ***, *p* < 0.0005; ###, *p* < 0.0005 compared between Altromin and Ssniff

Figure 2 shows the results of the CGRP release experiments, including two basal release values followed by stimulated CGRP release. For clarity, the release data were separately displayed for Altromin and Ssniff diet (Fig. 3A, B) and subdivided according to control and high-histamine groups and sexes. Analysing all data with the factors “diet” (Altromin vs. Ssniff), “histamine” (control vs. test groups) and “sex” (males vs. females), repeated measures ANOVA indicated highly significant changes in CGRP release in the course of the experiment (*F*_2,224_ = 620.80, *p* < 5*10^− 7^), as expected, but also between animals fed Altromin and animals fed Ssniff (*F*_1,112_ = 26.01, *p* < 5*10^− 6^), as well as between the sexes (*F*_1,112_ = 21.01, *p* < 5*10^− 5^), but not between the control and high-histamine diets (*F*_1,112_ = 0.86, *p* = 0.36). Generally, stimulated CGRP release seemed to be higher in samples of mice supplied with Altromin diet compared to mice supplied with Ssniff diet; also in samples of male compared to female Altromin diet fed mice (Fig. 3A, B). Post-hoc analysis with the Tukey HSD test showed no significant differences in basal release values between any of the groups, while the increase in stimulated CGRP release compared to basal release values was highly significant in all groups, as expected (*p* < 5*10^− 5^). In addition, the stimulated release values were significantly different between sexes in the Altromin diet groups and between the male Altromin diet and the male Ssniff diet group (Fig. 3 and Supplementary Table 2).

**Fig. 4:**
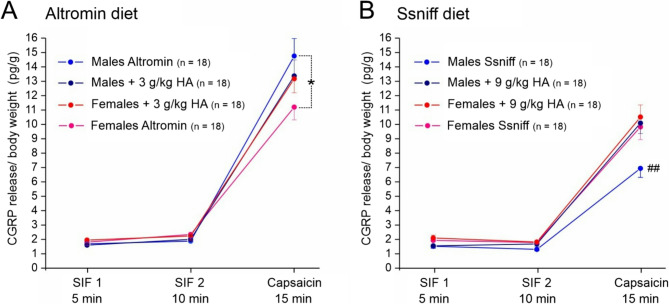
CGRP concentrations calculated relative to the body weight in hemisected skull preparations from animals fed with Altromin control diet or Altromin plus 3 g/kg histamine **A** and Ssniff control diet or Sniff plus 9 g/kg histamine **B**. Values are compared with repeated measures ANOVA followed by the Tukey post-hoc test and shown as means ± SEM; *, *p* < 0.05; ##, *p* < 0.005 compared between Altromin and Ssniff

Assuming that the sex difference may result from the different size of the animal’s heads and the dural surfaces, respectively, we also calculated the CGRP release relative to the individual body weight of the animals as a rough compensation for the different mass of the dura mater (Fig. 4A, B). Repeated measures ANOVA showed that the difference between Altromin and Ssniff diet was preserved (*F*_1,112_ = 23.97, *p* < 5*10^− 6^), while the sex difference was no longer significant (*F*_1,112_ = 0.46, *p* = 0.50), and there was a trend towards higher stimulated CGRP release values in high-histamine diet groups compared to control diet groups (*F*_1,112_ = 2.24, *p* = 0.14).

Post-hoc analysis with the Tukey HSD test showed that stimulated release values were significantly different between sexes in mice supplied with Altromin control diet and between the Altromin control diet and the Ssniff control diet group in males, but there was no significant difference between any of the control and high-histamine groups (Fig. 4 and Supplementary Table 3).

### Correlation of CGRP and Histamine release

**Fig. 5:**
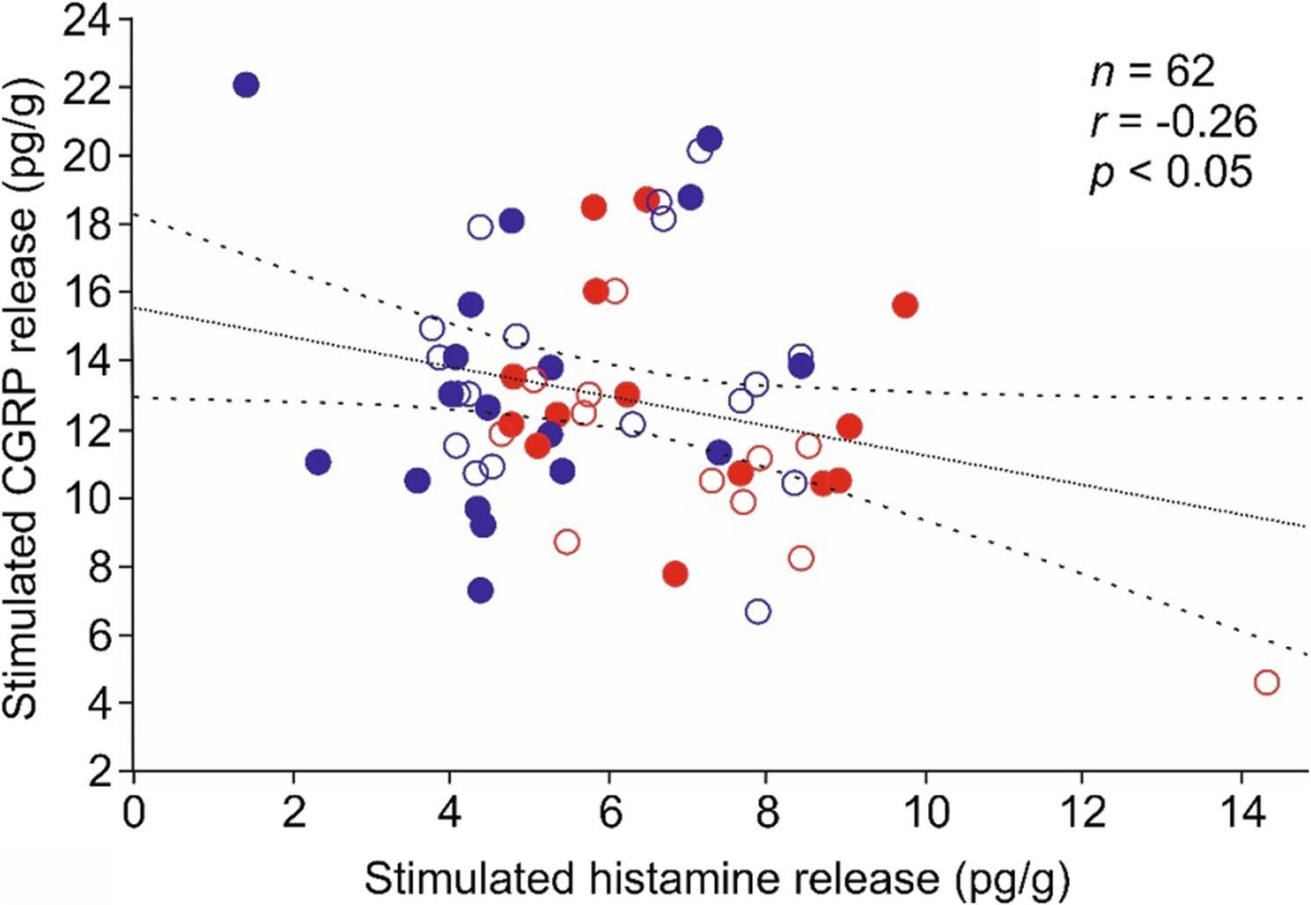
Linear correlation of CGRP release stimulated by capsaicin with histamine release stimulated by compound 48/80 in 62 hemi-skull preparations, normalized to the body weight of animals. Open circles denote preparations from animals fed Altromin control diet, filled circles Altromin plus 3 g/kg histamine. Blue circles denote preparations from male, red circles from female animals. Broken lines confine the 95% confidence interval

In this post-hoc analysis, data from same experiments in 62 hemi-skulls from 31 animals supplied with Altromin control diet or Altromin plus 3 g/kg histamine (high histamine diet), in which CGRP release was followed by histamine release, were subjected to pairwise correlation analysis. There was no correlation of basal CGRP and histamine release without stimulation (*n* = 62, product-momentum correlation, *r* = 0.02 for SIF 1, *r* = 0.00 for SIF2) but a significant negative correlation between CGRP release stimulated by capsaicin and histamine release stimulated by compound 48/80 (*r* = −0.26, *p* < 0.05; Fig. 5). This negative correlation was entirely attributable to female animals (*n* = 26, *r* = −0.48, *p* < 0.05), while it was not seen in the male samples (*n* = 36, *r* = 0.02). When separating the data between low and high histamine diet (irrespective of Altromin or Ssniff), there was again no correlation in the basal release values, however, CGRP and histamine release was negatively correlated in the experiments of animals with control diet (*n* = 30, *r* = −0.45, *p* < 0.05) but not with high histamine diet (*n* = 32, *r* = −0.04).

## Discussion

### CGRP content in different tissues

In order to determine a possible impact of dietary histidine/histamine intake on the production and distribution of CGRP in the body, the concentration of the neuropeptide was assessed in various organs. In all organs of animals supplied with Ssniff, the CGRP concentration was higher compared to animals supplied with Altromin (see Fig. 1). The trend towards higher CGRP concentrations in the gut of animals supplied with Sniff than with Altromin diet is interesting, because Ssniff diet overall bears a higher histamine/histidine content. We have first used Altromin C1072 food, because it contains only little histidine (4.675 mg/kg), whereas Ssniff contains over a thousand times more (5.8 g/kg). Histidine is likely converted into histamine by bacteria in the gut [1, 13] as well as in enterochromaffin-like cells of the stomach, and in mast cells [14]. We speculate that a difference in dietary histidine of this order of magnitude may be the main contributor to the generally higher CGRP content in all tissues of animals supplied with Ssniff compared to Altromin as a result of the local conversion into histamine. Thus adding histamine in the tested amounts was insufficient to elicit consistent systemic effects, likely because of rapid local degradation or other factors. The Altromin or Ssniff high-histamine diets likewise appeared inadequate to alter CGRP levels in plasma or neuronal tissues. This suggests that much higher dietary histamine would be needed to affect CGRP, while exogenous histamine generated from dietary histidine conversion may indeed influence CGRP.

The CGRP concentration in tissues innervated by trigeminal afferents (trigeminal ganglion and medulla oblongata containing the spinal trigeminal nucleus) is much higher than in other organs like the gut and the cerebellum. This is not surprising, since trigeminal peptidergic afferents produce high amounts of CGRP, which are released in the venous blood stream. In the cerebellum we have measured very low CGRP concentrations, which is surprising, because CGRP immunoreactive neurons have been described in the cerebellar cortex [15]. The differences in cerebellar CGRP concentrations between Altromin and Ssniff fed mice reflect rather the differences in plasma, assuming that the CGRP found here was from the remaining plasma in the cerebellar vessels, which have not been perfused during preparation (see Fig. 1). With some limitations this could also be true for the gut, which is highly vascularized but also innervated by neurons producing mainly β-CGRP [16], which is measured by our ELISA, like circulating α-CGRP. However, the number of trials was too low to draw a definitive conclusion.

### Correlation of Histamine plasma levels with CGRP concentrations in plasma and neural tissues

There was a tendency towards a positive correlation between CGRP and histamine concentration in plasma (see Fig. 2; Table 2). This may argue for an increased ongoing CGRP release in individual animals with higher plasma histamine levels. Our recently published data using the same high histamine-fed mouse model clearly demonstrated that dietary histamine and/or most likely histidine, significantly influence plasma histamine levels [10]. Notably, supplementation with 3 g/kg of histamine in the Altromin diet for 22–32 days resulted in a significant increase in plasma histamine, whereas a 3-day exposure, although showing a trend, did not reach statistical significance. Furthermore, the Ssniff diet - containing an even higher histamine concentration (9 g/kg) and highly elevated histidine levels - led to greater histamine diffusion into the bloodstream. Based on these findings, we hypothesized that correlating diet-derived plasma histamine levels with CGRP content in the trigeminal ganglion would offer a direct measure of the impact of gut histamine on neural CGRP expression. Indeed, correlation analysis revealed that elevated plasma histamine, primarily driven by histamine diffusion from the gastrointestinal system, was associated with increased CGRP production by trigeminal ganglion neurons. Given the established role of these neurons in migraine pathophysiology, this mechanism may explain how highly elevated histamine levels in the gut, whether present as histamine in the diet or rather converted from dietary histidine to histamine locally, contributes to migraine attacks, particularly in individuals with impaired diamine oxidase. A positive correlation was also found between histamine plasma levels and CGRP concentrations in the medulla oblongata (see Table 3), i.e., overall in the tissues innervated by CGRP releasing primary afferents. On the other hand, a tendency towards a negative correlation between CGRP and histamine concentration was seen in these neural tissues (see Table 2). Therefore, it may be speculated that high histamine plasma levels due to high dietary histidine/histamine intake have some sensitizing effects on neural tissues resulting in more CGRP production and more constitutive CGRP release, which may be followed by a tendency of higher histamine release from mast cells and hence decreased histamine concentrations in these tissues. According to our previous experiments, high dietary histidine/histamine was also reflected by significantly higher histamine concentrations in the gut [3]. However, no correlation was found between plasma histamine levels and the CGRP concentration in the gut (see Table 3) rendering a direct effect of histamine on the CGRP production in the enteric nervous system unlikely.

### CGRP release from the dura mater and sex differences

In order to determine a possible impact of dietary histamine/histidine on the CGRP release from the cranial dura mater, we measured basal release and then stimulated the dura with the potent TRPV1 agonist capsaicin to measure stimulated CGRP release from meningeal afferents in the hemisected skull from animals fed with Altromin or Ssniff diet, respectively, containing no or additional histamine. The basal CGRP release was not different between low and high histamine diet groups and between sexes. The stimulated release was more pronounced in all groups fed with Altromin diet compared to Sniff diet and significantly different between the two control diet groups in males (see Fig. 3). In addition, it was also different between the sexes in all Altromin diet groups. These differences were largely preserved when the data were normalized to the body weight of animals to compensate for the different head sizes and dural surfaces, provided that the innervation density of peptidergic trigeminal afferents is roughly constant (see Fig. 4).

The sex difference is not likely due to sex-specific differences in the sensitivity to capsaicin, which may even be higher in female animals due to estrogen-driven effects [17]. In contrast to this result, both the basal histamine release and the histamine release stimulated by the mast cell degranulator, compound 48/80, was higher in female animals, which was compensated, however, after normalizing to the body weight [3]. Generally, hormonal factors may influence digestion and histamine metabolism, which was not controlled in the present study. We have not checked the hormonal state of female animals, because due to the short oestrus cycle of the animals it changes several times during the exposure period to the diets. The preponderance in male animals regarding increased CGRP release after high dietary histamine may be interesting in terms of clinical comparisons of histamine-driven detrimental effects such as the prevalence of exogenous histamine-induced headache and migraine, in particular in DAO-deficient individuals. The predominance of CGRP-stimulated release in males following high dietary histamine/histidine intake raises the question of whether male mice may express lower levels of DAO compared to females, potentially rendering them more susceptible to the harmful effects of exogenous histamine in a sex-dependent manner. However, background evidence does not support a consistent imbalance in plasma or tissue DAO expression between sexes. One study reported significantly lower plasma DAO activity in healthy men compared to women [18], which could be relevant for our findings, provided that this is similar in rodents. Nonetheless, such a marked difference in DAO activity has not been consistently observed. For instance, a study in a Chilean population found a slightly lower, though non-significant, DAO activity in men, despite higher enzyme concentrations [19]. This trend was not observed in newborns [20], and a tendency toward lower levels being more common in women was also reported [21].

The stimulated CGRP release was significantly higher in male animals. The interpretation of these experimental results is limited regarding the higher prevalence of migraine in female patients [22, 23]. However, the sex difference in migraine prevalence is likely to depend more on hormone-dependent CGRP receptor expression and sensitivity than on higher CGRP levels in women compared to men [24, 25]. The stimulated CGRP release is generally increased when the dura mater is incubated with agents activating and sensitizing trigeminal afferents such as inflammatory mediators or nitric oxide donors [26, 27]. Thus the present result may argue for a sensitized state of meningeal afferents caused by long-term exposure of the body to high levels of dietary (exogenous) histidine/histamine. Possible functional connections of dietary histamine to the trigeminal system have been discussed recently [2] and warrant further research.

### Correlation of stimulated CGRP release with stimulated histamine release

The finding of a negative correlation between stimulated CGRP release and stimulated histamine release, especially in samples of female animals (see Fig. 4), was unexpected at first view. We cannot exclude that it was caused by our experimental procedure, in which we measured CGRP release followed by histamine release after extensive washing. High concentrations of CGRP have been found to release histamine from mast cells in the rodent dura mater [28, 29]. Thus higher CGRP release could have released more histamine, so that less histamine was remaining to be released by the subsequent treatment with compound 48/80. Alternatively, as already discussed above, we propose that high dietary histidine/histamine followed by increased histamine plasma levels may cause sensitization of meningeal afferents, which leads to permanently higher CGRP release and hence mast cell degranulation over time, finally resulting in lower histamine release upon compound 48/80 stimulation.

## Conclusion

We had reported that diets with high histamine/histidine cause elevated histamine plasma levels in mice [3]. Now, we show that an order of magnitude of over a thousand times more histidine in the diet may be the main contributor to both the higher plasma histamine levels and, in turn, the increased CGRP levels in tissues. Most dietary histidine is likely converted into histamine in the gut. Hence the relative contribution of the extra histamine added to both Ssniff and Altromin diets is likey minor to both the increased plasma levels and to CGRP expression.

Translated into the clinical situation, exogenous histamine induced sensitization of trigeminal (and other) primary afferents to generate CGRP may subsequently lead to a decreased activation threshold and hence facilitation of headache generation and triggering of migraine attacks.

## Supplementary Information

Supplementary material 1.

Supplementary material 2.

## Data Availability

All data is provided within the manuscript. Further data underlying the presentation of this study are available on request from the corresponding author.
