# Supplementary material for: Histamine plasma levels from dietary histidine/histamine intake correlate with CGRP in trigeminal tissues
Source: J Headache Pain. 2025 Nov 13;26(1):258. doi: 10.1186/s10194-025-02178-x (PMC12616900; doi:10.1186/s10194-025-02178-x)
Supplement: Supplementary file 1 — Supplementary material 1. [file 10194_2025_2178_MOESM1_ESM.docx]

|  | Altrom M Control | Altrom F Control | Altrom M  High HA | Altrom F High HA | Ssniff M  Control | Ssniff F Control | Ssniff M High HA |
| --- | --- | --- | --- | --- | --- | --- | --- |
| Altrom F Control | < 0.0005 |  |  |  |  |  |  |
| Altrom M  High HA | 0.435 | < 0.0005 |  |  |  |  |  |
| Altrom F High HA | < 0.0005 | 0.993 | < 0.005 |  |  |  |  |
| Ssniff M  Control | < 0.0005 | 0.998 | < 0.0005 | 0.318 |  |  |  |
| Ssniff F Control | < 0.0005 | 1.000 | < 0.0005 | 0.750 | 1.000 |  |  |
| Ssniff M High HA | < 0.0005 | 0.916 | 0.086 | 1.000 | 0.170 | 0.500 |  |
| Ssniff F High HA | < 0.00005 | 1.000 | < 0.0005 | 0.949 | 1.000 | 1.000 | 0.782 |

**Supplemental Table 1:** Significance values between stimulated CGRP release data analysed with the post-hoc Tukey HSD test following repeated measures ANOVA. Altrom, Altromin diet; Ssniff, Ssniff diet; M, males; F, females; HA, histidine/histamine.

**Supplementary Table 2:** Significance values between stimulated CGRP release data calculated relative to the body weight and analysed with the post-hoc Tukey HSD test following repeated measures ANOVA. Altrom, Altromin diet; Ssniff, Ssniff diet; M, males; F, females; HA, histidine/histamine.

|  | Altrom M Control | Altrom F Control | Altrom M  High HA | Altrom F High HA | Ssniff M  Control | Ssniff F Control | Ssniff M High HA |
| --- | --- | --- | --- | --- | --- | --- | --- |
| Altrom F Control | < 0.05 |  |  |  |  |  |  |
| Altrom M  High HA | 0.996 | 0.691 |  |  |  |  |  |
| Altrom F High HA | 0.978 | 0.835 | 1.000 |  |  |  |  |
| Ssniff M  Control | < 0.0005 | < 0.05 | < 0.005 | < 0.005 |  |  |  |
| Ssniff F Control | < 0.005 | 0.999 | 0.074 | 0.128 | 0.532 |  |  |
| Ssniff M High HA | < 0.05 | 1.000 | 0.153 | 0.244 | 0.353 | 1.000 |  |
| Ssniff F High HA | < 0.05 | 1.000 | 0.386 | 0.533 | 0.149 | 1.000 | 1.000 |
