# Supplementary material for: Histamine plasma levels from dietary histidine/histamine intake correlate with CGRP in trigeminal tissues
Source: J Headache Pain. 2025 Nov 13;26(1):258. doi: 10.1186/s10194-025-02178-x (PMC12616900; doi:10.1186/s10194-025-02178-x)
Supplement: Supplementary file 2 — Supplementary material 2. [file 10194_2025_2178_MOESM2_ESM.docx]

**Supplementary Table 3:** Significance values between CGRP concentrations found in plasma, ileum, trigeminal ganglia (TG) and cerebellum (Cereb) of mice fed with Altromin (Altrom) or Ssniff control or high-histamine (high HA) diet analysed with the post-hoc Tukey HSD test following factorial ANOVA

| **Plasma** | Altrom control | Altrom  high HA | Ssniff  control |
| --- | --- | --- | --- |
| Altrom  high HA | 0.999 |  |  |
| Ssniff  control | 0.046 | 0.063 |  |
| Ssniff high HA | 0.267 | 0.329 | 0.866 |

| **TG** | Altrom control | Altrom  high HA | Ssniff  control |
| --- | --- | --- | --- |
| Altrom  high HA | 0.968 |  |  |
| Ssniff  control | < 0.0005 | < 0.0005 |  |
| Ssniff high HA | < 0.0005 | < 0.0005 | 0.312 |

| **Cereb** | Altrom control | Altrom  high HA | Ssniff  control |
| --- | --- | --- | --- |
| Altrom  high HA | 0.951 |  |  |
| Ssniff  control | < 0.0005 | < 0.0005 |  |
| Ssniff high HA | < 0.0005 | < 0.0005 | 1.000 |

| **Ileum** | Altrom control | Altrom  high HA | Ssniff  control |
| --- | --- | --- | --- |
| Altrom  high HA | 0.999 |  |  |
| Ssniff  control | 0.395 | 0.460 |  |
| Ssniff high HA | 0.007 | 0.009 | 0.152 |
